# Retraction: Electro-oxidation reconstitution of aluminium copper MOF-derived metal oxyhydroxides for a robust OER process

**DOI:** 10.1039/d6ra90165d

**Published:** 2026-11-09

**Authors:** H. A. Alburaih, Sumaira Manzoor, M. Abdullah, M. N. Ashiq, Salma Aman, Sergei V. Trukhanov, Tatiana I. Zubar, Zhipeng Sun, T. A. Taha, Alex V. Trukhanov

**Affiliations:** a Department of Physics, College of Science, Princess Nourah bint Abdulrahman University P.O. Box 84428 Riyadh 11671 Saudi Arabia; b Institute of Chemical Sciences, Bahauddin Zakariya University Multan 60800 Pakistan; c Department of Chemistry, Government College University Lahore Pakistan; d Smart Sensors Laboratory, Department of Electronic Materials Technology, National University of Science and Technology MISiS Moscow 119049 Russia; e Scientific-Practical Materials Research Centre of National Academy of Sciences of Belarus Minsk 220072 Belarus fix.tatyana@gmail.com; f School of Materials and Energy, Guangdong University of Technology, Guangzhou Higher Education Mega Center No. 100 Waihuan Xi Road, Panyu District Guangzhou 510006 P. R. China; g Physics Department, College of Science, Jouf University P.O. Box 2014 Sakaka Saudi Arabia; h Physics and Engineering Mathematics Department, Faculty of Electronic Engineering, Menoufia University Menouf 32952 Egypt

## Abstract

Retraction of ‘Electro-oxidation reconstitution of aluminium copper MOF-derived metal oxyhydroxides for a robust OER process’ by H. A. Alburaih *et al.*, *RSC Adv.*, 2023, **13**, 8736–8742, https://doi.org/10.1039/d2ra07661f.

The Royal Society of Chemistry hereby wholly retracts this *RSC Advances* article due to concerns with the reliability of the data.

Concerns were raised with the XRD data in Fig. 1. The authors provided what they stated to be the raw data; however, an independent expert concluded that the datasets contain features inconsistent with genuine experimental XRD measurements and are unlikely to have arisen from genuine experimental measurements.

Given the significance of these concerns, the Editor has lost confidence that the findings presented in this paper are reliable.

We attempted to inform all authors about the retraction of the article. Author T. A. Taha was not reachable, and the other authors have not responded.

Signed: Laura Fisher, Executive Editor, *RSC Advances*

Date: 23rd October 2026

